# Use of Small-Area Estimates to Describe County-Level Geographic Variation in Prevalence of Extreme Obesity Among US Adults

**DOI:** 10.1001/jamanetworkopen.2020.4289

**Published:** 2020-05-08

**Authors:** Carrie W. Mills, Glen Johnson, Terry T. K. Huang, Deborah Balk, Katarzyna Wyka

**Affiliations:** 1 Center for Systems and Community Design, The City University of New York Graduate School of Public Health & Health Policy, New York, New York; 2 CUNY Institute for Demographic Research, The City University of New York, New York, New York; 3 Marxe School of Public and International Affairs, Baruch College, The City University of New York, New York, New York

## Abstract

**Importance:**

The prevalence of extreme obesity continues to increase among adults in the US, yet there is an absence of subnational estimates and geographic description of extreme obesity. This shortcoming prevents a thorough understanding of the geographic distribution of extreme obesity, which in turn limits the ability of public health agencies and policy makers to target areas with a known higher prevalence.

**Objectives:**

To use small-area estimation to create county-level estimates of extreme obesity in the US and apply spatial methods to identify clusters of high and low prevalence.

**Design, Setting, and Participants:**

A cross-sectional analysis was conducted using multilevel regression and poststratification with data from the 2012 Behavioral Risk Factor Surveillance System and the US Census Bureau to create prevalence estimates of county-level extreme obesity (body mass index ≥40 [calculated as weight in kilograms divided by height in meters squared]). Data were included on adults (aged ≥18 years) living in the contiguous US. Analysis was performed from June 4 to December 28, 2018.

**Main Outcomes and Measures:**

Multilevel logistic regression models estimated the probability of extreme obesity based on individual-level and area-level characteristics. Census counts were multiplied by these probabilities and summed by county to create county-level prevalence estimates. Moran index values were calculated to assess spatial autocorrelation and identify spatial clusters of hot and cold spots. Estimates of moderate obesity were obtained for comparison.

**Results:**

Overall, the weighted prevalence of extreme obesity was 4.0% (95% CI, 3.9%-4.1%) and the prevalence of moderate obesity was 23.7% (95% CI, 23.4%-23.9%). County-level prevalence of extreme obesity ranged from 1.3% (95% CI, 1.3%-1.3%) to 15.7% (95% CI, 15.3%-16.0%). The Pearson correlation coefficient comparing model-predicted estimates with direct estimates was 0.81 (*P* < .001). The Moran index *I* score was 0.35 (*P* < .001), indicating spatial clustering. Significant clusters of high and low prevalence were identified. Hot spots indicating clustering of high prevalence of extreme obesity in several regions, including the Mississippi Delta region and the Southeast, were identified, as well as clusters of low prevalence in the Rocky Mountain region and the Northeast.

**Conclusions and Relevance:**

Substantial geographic variation was identified in the prevalence of extreme obesity; there was considerable county-level variation even in states generally known as having high or low prevalence of obesity. The results suggest that extreme obesity prevalence demonstrates spatial dependence and clustering and may support the need for substate analysis and benefit of disaggregation of obesity by group. Findings from this study can inform local and national policies seeking to identify populations most at risk from very high body mass index.

## Key Points

**Question:**

What is the county-level prevalence of extreme obesity in the United States?

**Findings:**

In this cross-sectional study of adults in the United States, county-level prevalence of self-reported extreme obesity ranged from 1.3% to 15.7%. Several prominent clusters of high prevalence were identified, including in the Mississippi Delta and the Southeast.

**Meaning:**

Prevalence of extreme obesity appears to vary considerably by county; heterogeneity is obscured by available state-level prevalence estimates.

## Teaser

This cross-sectional study estimates county-level rates of extreme obesity identifies clusters of high and low prevalence within the US.

## Introduction

As the obesity epidemic enters its fifth decade in the US, estimates indicate that the prevalence of obesity among adults has reached 37.7%.^1^ Furthermore, the population with class III, or extreme, obesity (body mass index [BMI] ≥40 [calculated as weight in kilograms divided by height in meters squared]), has increased from 2.9% in 1988-1994 to 7.7% in 2015-2016.^2,3^ The high rate of extreme adult obesity is likely to not only continue but to increase. The annual incidence of extreme obesity was most recently estimated to be 0.7%.^4^ While obesity overall has been the focus of extensive research, much less is known about extreme obesity. Given the increasing prevalence of extreme obesity, it is necessary to disaggregate obesity to better understand differences in both the epidemiologic factors and morbidity and mortality by class of obesity.^5^

Existing research on extreme obesity describes variation in prevalence by sex, race/ethnicity, and other demographic factors.^1,2,6,7,8,9,10^ In contrast, patterns of geographic variation appear to be missing, which limits the ability of public health agencies and policy makers to target areas with a known higher prevalence.

Currently, surveys designed and weighted for national- or state-level estimates are the primary source of data on obesity surveillance.^11^ State estimates, however, often mask the heterogeneity of health risk differences within communities because of regional differences in population age distribution and socioeconomic factors, such as race/ethnicity and poverty; this heterogeneity has been demonstrated in obesity research.^12^ Statistical methods for small-area estimation, ie, for providing prevalence estimates at a substate level, include multilevel regression and poststratification (MRP), which combines estimates from a multilevel prediction model and stratified population counts to estimate desired prevalence.^13,14,15^

Small-area estimation methods have been used to create county-level prevalence estimates of obesity,^12,16^ but, to our knowledge, similar county-level estimates by class of obesity have never been published or publicly estimated. The aim of our study was to create prevalence estimates of county-level extreme obesity using MRP and assess spatial clustering of prevalence estimates. To facilitate comparison between extreme obesity and obesity in general, this analysis also calculated estimates for moderate obesity.

## Methods

### Data Sources

We conducted a retrospective, cross-sectional observational study using the 2012 Behavioral Risk Factor Surveillance System (BRFSS) and data from the US Census Bureau. The BRFSS is an annual telephone survey of adults aged 18 and older conducted by the Centers for Disease Control and Prevention to monitor health-related behavior of the noninstitutionalized US population and was used to obtain individual-level variables used in the MRP prediction model. The cross-sectional survey is weighted to allow for direct national and state-level estimates.^17^ The 2012 survey was chosen because a county-level residence indicator is not available from 2013 onward.

County-level population counts for each of the 3109 counties in the contiguous US were obtained from the 2010 US Census for the purpose of weighting estimates for the MRP. County-level covariates used in the MRP prediction model were obtained from the 2012 American Community Survey 5-year roll-up. The American Community Survey, conducted by the US Census Bureau, is an annual nationwide survey that provides detailed information about select social, economic, and housing characteristics of the US population.^18^ This study is reported following the Strengthening the Reporting of Observational Studies in Epidemiology (STROBE) reporting guideline for cross-sectional studies.^19^ The CUNY School of Public Health Human Research Protection Program indicated that institutional review board approval was not required because secondary analysis of publicly available and deidentified data is not human subjects research.

### Outcome Variable

The primary outcome was county-specific prevalence estimates of extreme obesity. Body mass index was calculated from self-reported weight and height. Extreme obesity (yes/no) was defined as BMI greater than or equal to 40.0. A secondary outcome of moderate obesity (yes/no) was defined as BMI between 30.0 and 39.9. The public-use BRFSS data set excluded BMI for pregnant women (n = 2873). Individual-level covariates included sex (male/female), age group (18-24, 25-29, 30-34, 35-39, 40-44, 45-49, 50-54, 55-59, 60-64, 65-69, 70-74, 75-79, and ≥80 years), and race/ethnicity (non-Hispanic white, non-Hispanic black, Hispanic, Asian, Hawaii Native/Pacific Islander, American Indian/Alaska Native, other single race, and multiracial). County-level sociodemographic characteristics were the percentage of county residents living below the federal poverty level, the percentage of adults aged 25 years or older with a bachelor’s degree, and the percentage of county residents who lived in rural settings, since these factors are understood to be associated with obesity.^20,21^ County-level variables were categorized into quartiles, based on the national distribution. Finally, county-level census counts stratified by age group × sex × race/ethnicity were used in the poststratification step to obtain estimates. Cross-tabulated fields for age group × sex × race/ethnicity (208 groups) were extracted at the county level, and categorization for each demographic identifier was identical to categories used in the BRFSS survey data.

### Statistical Analysis

Before model construction, counties missing any survey data owing to sampling variation and state-specific determination of substate sampling^17^ (878 of 3109 counties) were aggregated to larger countylike areas. Neighboring counties were joined using an iterative aggregation tool^22^ until a minimum of 5 survey observations were obtained, resulting in 2215 county or countylike areas (eMethods 1 in the Supplement), referred to henceforth as *counties*. Survey responses, population counts, and covariate data were summed across aggregated counties. County-level prevalence was then obtained through a 2-step process, as follows.

First, a multilevel logistic regression model was fit to estimate the probability of extreme obesity using BRFSS data, accounting for both individual-level and county-level covariates.^23^ Random effects for county and state were included in the model to allow each to represent the association of county and state contextual effects with the outcome, accounting for between-area variation that cannot be explained with the inclusion of ancillary variables.^24^ Survey weights were rescaled by state to ensure accurate parameter SEs from model results^23^ and a weight statement was included in the model. Covariates that were not significant at *P* < .05 with 2-tailed testing were excluded and the model was refit. Educational level was the only county-level variable that was significantly associated with extreme obesity in the multivariable models. The fitted model was applied to estimate the average probability of extreme obesity for each cross classification of characteristics, county, and state (eMethods 2 in the Supplement).

In the second step, parameter estimates were applied to corresponding census population counts to obtain the prevalence of extreme obesity weighted to the demographic characteristics of the county’s population. Model parameter estimates were summed for each of the 208 age groups × sex × race/ethnicity × county groupings and then multiplied by the population count for the corresponding age group × sex × race/ethnicity within the county (eMethods 3 and eFigure 1 in the Supplement). After weighting the predicted value by the actual subgroup proportion within each county for each of the aforementioned cells, all cells within a county were summed to produce the county-level prevalence estimates.^23^ After repeating these steps with moderate obesity as the outcome, choropleth maps were created for both extreme and moderate obesity estimates for counties, using 5 classes based on quantile breaks.^25^

Overall spatial dependence for the prevalence of each obesity group was assessed using the global Moran index *I* test, which compares neighboring units across the whole study area to inform positive spatial autocorrelation or dispersion. A positive value of the Moran index *I* statistic (range, −1 to 1) indicates clustering, a negative value indicates dispersal, and 0 indicates complete spatial randomness (eMethods 4 in the Supplement). A *P* value was calculated for each indicator by running 999 permutations to create a distribution around the original index value.

Local indicators of spatial association were used to identify areas with local spatial clustering (eMethods 4 in the Supplement).^26,27^ This method computes county-specific Moran index *I* statistics based on a local neighborhood of counties, which we defined through a first-order queen contiguity specification. Alternative specifications were assessed in sensitivity analyses (eFigure 2 in the Supplement). County-specific local Moran index *I* statistics were mapped to show significant clusters of obesity group prevalence, identifying hot spots (high-high [clustering of high prevalence]) and cold spots (low-low [clustering of low prevalence]), as well as counties classified as low-high, indicating counties with low prevalence adjacent to those with high prevalence, and the inverse scenario of high-low.

Internal validation examined the degree to which model estimates correlated with known data estimates in 2 ways: first, model-predicted estimates created by MRP were compared with direct unweighted estimates from all counties with 100 or more survey observations^14,23^; second, county-level estimates were aggregated to yield state estimates using a population-weighted average and compared with direct state-level weighted survey estimates.^28^ Prevalence estimates by state were calculated accounting for the complex sampling design of the BRFSS. In both scenarios, descriptive statistics, including median, range, and interquartile range, were compared and Pearson correlation coefficients were computed to assess linear correlation between the 2 sets of estimates. Each of these methods was performed separately for moderate and extreme obesity.

Data analysis was performed from June 4 to December 28, 2018, using SAS, version 9.4 (SAS Institute Inc), GeoDa, version 1.8.16.4,^29^ and QGIS, version 2.18.

## Results

Overall, the weighted prevalence of extreme obesity was 4.0% (95% CI, 3.9%-4.1%) and the prevalence of moderate obesity was 23.7% (95% CI, 23.4%-23.9%). Weighted state prevalence of extreme obesity ranged from 2.5% (95% CI, 2.1%-2.9%) in Colorado to 6.1% (95% CI, 5.3%-6.9%) in Louisiana (Table 1). Prevalence of moderate obesity ranged from 18.0% (95% CI, 17.1%-18.9%) in Colorado to 28.9% (95% CI, 27.4%-30.4%) in Mississippi. Weighted prevalence estimates by demographic factor can be found in the eTable in the Supplement.

**Table 1.** Weighed Prevalence Estimates of Extreme and Moderate Obesity by State, United States, BRFSS 2012^a^

State	Obesity, % (95% CI)	
	Extreme	Moderate
Alabama	5.7 (5.0-6.4)	27.3 (25.9-28.6)
Alaska	3.8 (2.9-4.7)	21.9 (20.2-23.6)
Arizona	3.3 (2.5-4.0)	22.7 (21.1-24.4)
Arkansas	5.8 (4.9-6.8)	28.7 (26.9-30.4)
California	3.2 (2.7-3.7)	21.8 (20.8-22.8)
Colorado	2.5 (2.1-2.9)	18.0 (17.1-18.9)
Connecticut	3.3 (2.8-3.9)	22.2 (21.0-23.5)
Delaware	3.7 (2.9-4.4)	23.2 (21.6-24.8)
Florida	3.8 (3.0-4.5)	21.4 (20.0-22.9)
Georgia	4.3 (3.6-5.1)	24.8 (23.2-26.4)
Hawaii	2.9 (2.3-3.6)	20.6 (19.1-22.1)
Idaho	3.4 (2.7-4.2)	23.4 (21.4-25.3)
Illinois	4.6 (3.7-5.4)	23.6 (22.0-25.2)
Indiana	4.7 (4.2-5.3)	26.6 (25.4-27.9)
Iowa	4.6 (3.9-5.2)	25.8 (24.6-27.1)
Kansas	4.6 (4.1-5.1)	25.2 (24.2-26.3)
Kentucky	5.4 (4.7-6.0)	25.9 (24.6-27.2)
Louisiana	6.1 (5.3-6.9)	28.6 (27.1-30.2)
Maine	4.3 (3.7-4.9)	24.0 (23.0-25.1)
Maryland	3.7 (3.1-4.2)	23.9 (22.7-25.2)
Massachusetts	2.7 (2.4-3.1)	20.2 (19.3-21.0)
Michigan	5.0 (4.4-5.5)	26.1 (24.9-27.3)
Minnesota	3.0 (2.6-3.4)	22.7 (21.7-23.7)
Mississippi	5.7 (5.0-6.5)	28.9 (27.4-30.4)
Missouri	4.8 (4.0-5.5)	24.8 (23.3-26.3)
Montana	3.7 (3.1-4.2)	20.6 (19.5-21.8)
Nebraska	3.8 (3.4-4.2)	24.8 (23.9-25.7)
Nevada	3.4 (2.6-4.2)	22.8 (21.0-24.6)
New Hampshire	4.2 (3.5-4.9)	23.1 (21.7-24.5)
New Jersey	2.8 (2.4-3.2)	21.8 (20.8-22.8)
New Mexico	3.5 (3.0-4.0)	23.6 (22.4-24.8)
New York	2.8 (2.3-3.4)	20.7 (19.3-22.2)
North Carolina	4.3 (3.8-4.8)	25.3 (24.2-26.3)
North Dakota	3.4 (2.7-4.1)	26.3 (24.6-28.0)
Ohio	5.0 (4.5-5.5)	25.1 (24.0-26.2)
Oklahoma	5.0 (4.3-5.6)	27.2 (25.9-28.6)
Oregon	4.2 (3.4-4.9)	23.1 (21.6-24.7)
Pennsylvania	4.3 (3.9-4.8)	24.7 (23.8-25.7)
Rhode Island	2.8 (2.2-3.4)	22.9 (21.4-24.5)
South Carolina	5.1 (4.6-5.7)	26.4 (25.3-27.6)
South Dakota	3.5 (2.9-4.1)	24.6 (23.1-26.2)
Tennessee	5.0 (4.3-5.7)	26.1 (24.7-27.6)
Texas	4.1 (3.5-4.6)	25.1 (23.8-26.4)
Utah	3.3 (2.9-3.7)	21.0 (20.0-21.9)
Vermont	3.2 (2.5-3.8)	20.6 (19.2-21.9)
Virginia	4.0 (3.4-4.6)	23.4 (22.1-24.7)
Washington	3.9 (3.5-4.4)	22.9 (21.9-23.8)
Washington, DC	3.0 (2.2-3.8)	18.9 (16.9-20.9)
West Virginia	5.5 (4.7-6.2)	28.3 (26.8-29.8)
Wisconsin	5.1 (4.2-6.0)	24.6 (22.8-26.4)
Wyoming	3.3 (2.6-4.0)	21.3 (19.6-23.0)

Abbreviation: BRFSS, Behavioral Risk Factor Surveillance System.

^a^ Includes data from all 50 states and Washington, DC.

**Figure 1.**
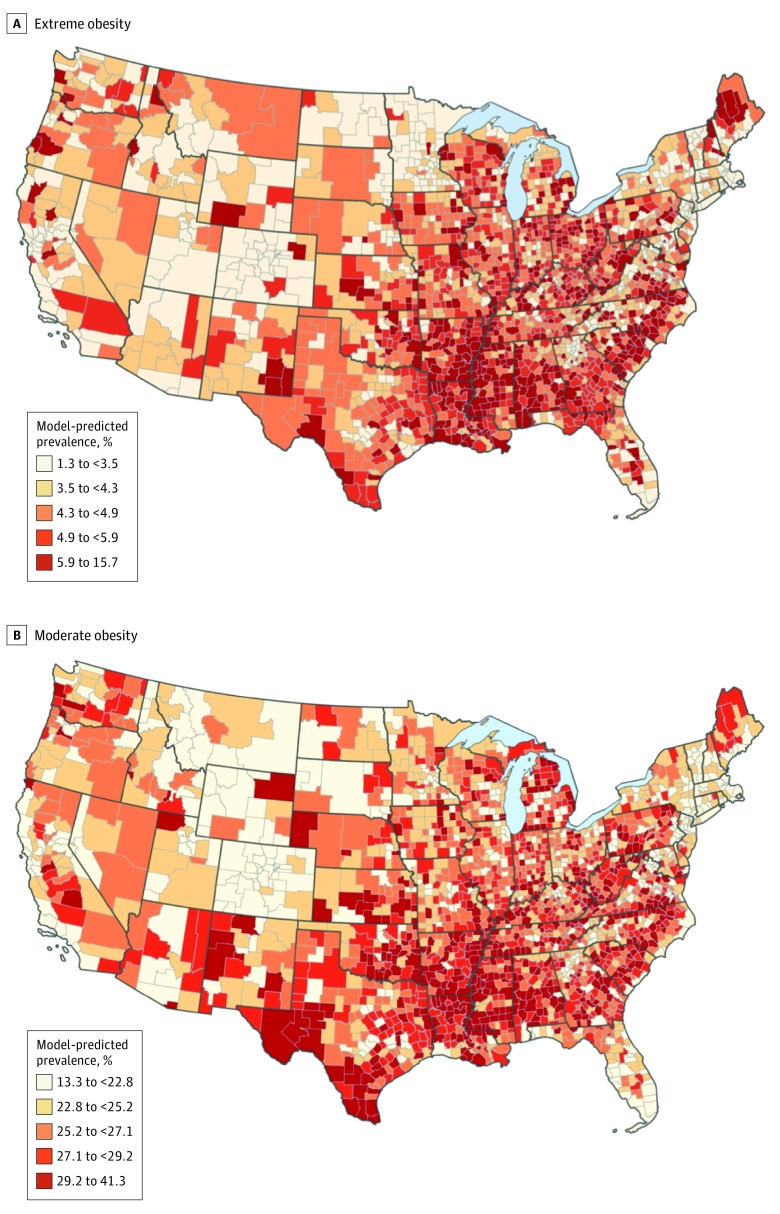
Model-Predicted Prevalence Estimates of Obesity Extreme (A) and moderate (B) obesity among adults by county, United States, 2012 Behavioral Risk Factor Surveillance System. Classes divided based on quantile breaks.

County-level prevalence estimates of extreme obesity ranged from 1.3% (95% CI, 1.3%-1.3%) to 15.7% (95% CI, 15.3%-16.0%), with a median of 4.6% (95% CI, 4.5%-4.7%) (Figure 1A; eFigure 3 in the Supplement). The highest prevalence of extreme obesity was found among counties in Ohio, Arkansas, and Alabama. Counties with the lowest prevalence of extreme obesity were found in Colorado, California, and Massachusetts. States that showed the greatest variability of county-level prevalence estimates of extreme obesity included Ohio, where estimates ranged from 3.0% (95% CI, 2.9%-3.0%) to 15.7% (95% CI, 15.3%-16.0%), followed by Arkansas (from 2.5%; 95% CI, 2.4%-2.6% to 15.1%; 95% CI, 14.8%-15.5%) and South Carolina (from 2.0%; 95% CI, 2.0%-2.0% to 12.2%; 95% CI, 11.9%-12.4%). In California, the most populous state, prevalence estimates ranged from 1.7% (95% CI, 1.7%-1.8%) to 8.8% (95% CI, 8.5%-8.9%), and in Texas, the largest state areawise, estimates ranged from 2.5% (95% CI, 2.4%-2.6%) to 7.4% (95% CI, 7.2%-7.5%).

The county-level prevalence of moderate obesity ranged from 13.3% (95% CI, 13.1%-13.4%) to 41.3% (95% CI, 41.0%-41.6%), with a median of 26.1% (95% CI, 25.9%-26.4%) (Figure 1B; eFigure 4 in the Supplement). The highest prevalence was found among counties in Louisiana, Mississippi, and Arkansas; the lowest prevalence was found in Colorado, New York, and California. States that showed the greatest variability of county-level prevalence estimates of moderate obesity included Idaho (from 14.7%; 95% CI, 14.6%-14.9%, to 37.5%; 96% CI, 37.2%-37.8%) followed by California (from 14.2%; 95% CI, 14.1%-14.4% to 34.6%; 95% CI, 34.4%-34.8%) and Louisiana (from 21.4%; 95% CI, 21.2%-21.5% to 41.3%; 95% CI, 41.0%-41.6%).

### Spatial Dependence and Clustering

The global Moran index *I* statistic for extreme obesity was 0.35 (*P* < .001), indicating that the distribution of prevalence of extreme obesity was spatially autocorrelated. The Moran index *I* statistic for moderate obesity was similar to that for extreme obesity, at 0.38 (*P* < .001). Given that one would expect a high level of heterogeneity at the county level, having this much spatial dependence is considered meaningful.

**Figure 2.**
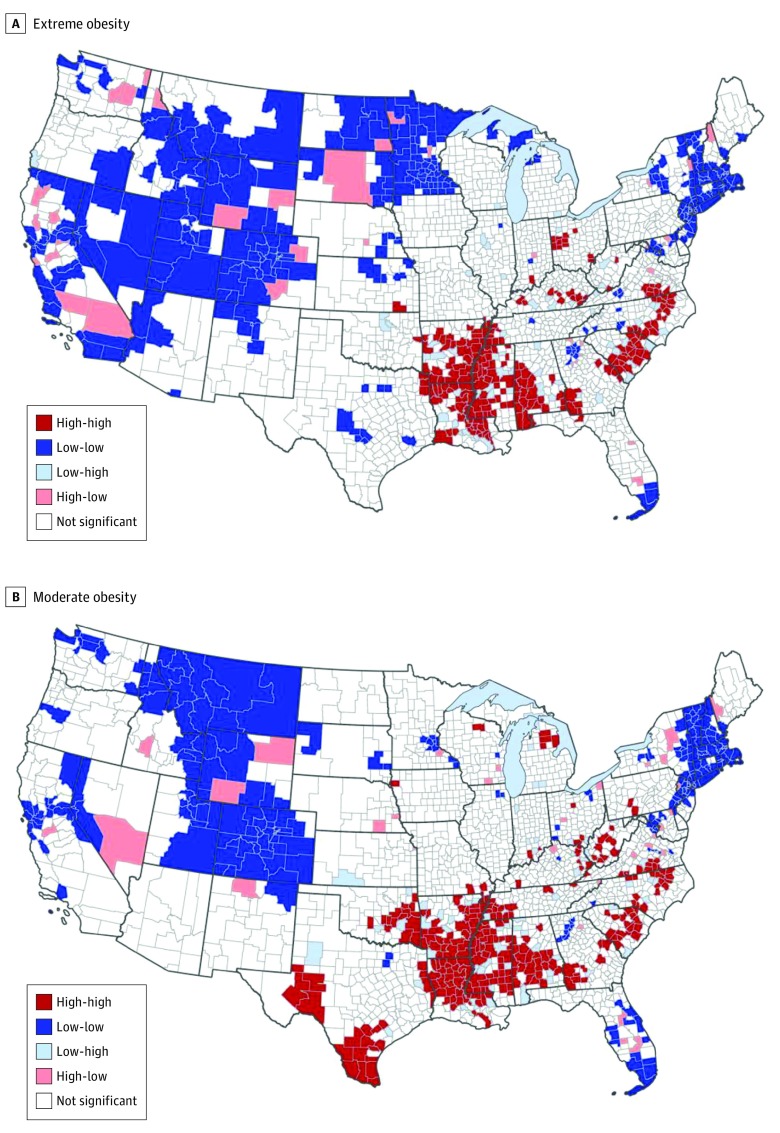
Local Indicators of Spatial Association Cluster Maps for Obesity Extreme (A) and (B) moderate obesity among adults by county in the contiguous United States, 2012. High-high indicates clustering of high prevalence; low-low, clustering of low prevalence; low-high, counties with low prevalence adjacent to those with high prevalence; and high-low, counties with high prevalence adjacent to those with low prevalence.

Statistically significant spatial clusters of extreme obesity are presented in Figure 2A. Of the 2215 counties, there were 208 hot-spot counties and 326 cold-spot counties. The largest cluster was in the Mississippi Delta region, composed of counties surrounding the Mississippi River in the Southeast and in parts of Texas. The second largest cluster was also in the Southeast, predominantly among counties in North Carolina and South Carolina and Virginia. Additional small hot spots were found among counties in Ohio’s rural northwest region and Kentucky.

A broad swath of the western US and the Great Plains region showed cold spots of a lower prevalence of extreme obesity. The second predominant cold spot was located among counties in New England. There were many additional small cold spots, including within Washington, DC, and surrounding suburbs, the southern tip of Florida, and central Texas. Georgia also contained a small cold-spot cluster of lower prevalence of extreme obesity in the counties comprising the Atlanta metropolitan area.

Statistically significant spatial clusters of moderate obesity are presented in Figure 2B. There were 262 hot-spot counties and 248 cold-spot counties. Similar to extreme obesity, the predominant cluster of moderate obesity was composed of counties in the southern states surrounding the Mississippi River. Unlike with extreme obesity, this cluster also included counties in the eastern part of Oklahoma. There were also 2 hot-spot clusters in west and south Texas. Similar to extreme obesity, there was a noticeable, but smaller, cluster of moderate obesity found in North Carolina, South Carolina, and Virginia. Additional small, less-dense clusters of moderate obesity were found in West Virginia, Ohio, Kentucky, and northern Michigan.

Relative to extreme obesity, a smaller area of the country showed cold spots for moderate obesity. In addition to the dominant western cold spot in Montana down through Utah and Colorado, the second cold spot for moderate obesity was located among counties in New England and was denser than for extreme obesity. Similar to extreme obesity, there was also a cold spot in Washington, DC, and surrounding suburbs. The southern tip of Florida also showed a cluster of lower prevalence, and this cluster was larger for moderate obesity than for extreme obesity and also extended into central Florida. The only area in Minnesota that was a cold spot was the Minneapolis-St Paul metropolitan area; this finding is in contrast to the entire state of Minnesota forming the majority of an extreme obesity cold-spot cluster.

### Sensitivity Analysis

There were 867 counties with 100 or more survey observations included in the first internal validation approach. The Pearson correlation coefficient, *r*, between direct unweighted prevalence estimates and model-predicted estimates was 0.81 (*P* < .001) for extreme obesity and 0.86 (*P* < .001) for moderate obesity (Table 2). The Pearson *r* value between weighted direct state-level estimates and model-predicted estimates based on aggregated county-level estimates (the second validation approach) was 0.99 (*P* < .001) for both extreme and moderate obesity.

**Table 2.** County-Level Estimates and Weighted State-Level Estimates of Prevalence of Extreme and Moderate Obesity Among Adults With Model-Predicted Estimates, United States, 2012 BRFSS^a^

Estimate	Correlation coefficient^b^	Prevalence estimate, %						
		Minimum	Quartile 1	Medium	Quartile 3	Maximum	IQR	Range
**Extreme obesity**								
Counties^c^								
Direct	0.81	0	2.7	3.9	5.1	13.6	2.4	13.6
SAE		1.3	3.2	4.2	5.2	12.2	2.0	10.9
States^d^								
Weighted direct	0.99	2.5	3.3	4.0	4.8	6.1	1.4	3.6
Aggregated SAE		2.6	3.4	4.0	4.8	5.9	1.4	3.3
**Moderate obesity**								
Counties^c^								
Direct	0.86	4.3	20.6	23.7	26.8	39.0	6.2	34.8
SAE		13.3	21.6	24.5	27.5	36.6	5.9	23.3
States^d^								
Weighted direct	0.99	18.0	22.7	23.9	25.8	28.9	3.1	10.9
Aggregated SAE		18.1	22.2	23.7	25.2	28.7	3.0	10.6

Abbreviations: BRFSS, Behavioral Risk Factor Surveillance System; IQR, interquartile range; SAE, small-area estimation.

^a^ Model containing sex, age group, race/ethnicity, educational level, and county and state random effects.

^b^ Pearson correlation coefficient.

^c^ Among 867 counties with 100 or more observations.

^d^ Among 48 contiguous states and Washington, DC (n = 49).

## Discussion

To our knowledge, this is the first study that estimated county-level prevalence of extreme obesity across the US. Results indicate that the prevalence of extreme obesity varied substantially among counties and states. The highest prevalence was most often found in counties in the southern US, while the lowest prevalence was most often in the northeastern and western regions. Mapping of prevalence showed similar overall patterns of extreme obesity and moderate obesity, with differences identified when the focus was more local. Estimates were found to be well validated in internal validity tests. Our findings appear to show that county-level prevalence of extreme obesity is spatially dependent in the contiguous US. We identified and located significant hot spots of extreme obesity, including 2 large hot spots in the southeastern states and a small cluster in Ohio. The prevalence of moderate obesity was also found to be spatially dependent, with hot spots similarly identified in the southeastern states as well as in Texas.

Our findings provide information about variation in the prevalence of extreme obesity. While state estimates have a 2- to 3-fold range (from 2.5% to 6.1%), counties showed much greater variability, particularly at the higher end, with a 12-fold range of estimates (from 1.3% to 15.7%). In addition, there appears to be substantial variation of county-level prevalence within states, even in states consistently showing higher rates of obesity, such as Oklahoma and Kentucky, and those showing lower rates of obesity, such as California.^30^ These findings reinforce the importance of examining prevalence at a substate level to identify the areas with the greatest burden and need. County-level estimates allow for state and local health departments to more clearly identify jurisdictions where public health interventions and efforts targeting extreme obesity may be most crucial.

In addition, our estimates appeared to successfully identify local differences. For example, the northwest tip of Arkansas shows a noticeably lower prevalence of extreme obesity than surrounding areas. This area, home to the Walmart world headquarters, has a different population structure than surrounding areas. Recent data indicate that, while 72% of adults in Arkansas are non-Hispanic white and 22% have a bachelor’s degree, 82% of those in the county where Walmart is located (Benton) are non-Hispanic white and more than 29% have a bachelor’s degree.^31^ The MRP estimates identified this difference through demographic data, leading to lower prevalence estimates.

While there was substantial overlap of areas with low and high prevalence of both moderate and extreme obesity, there were also some notable differences. Parts of Florida and Maine indicated some of the highest prevalence rates of extreme but not moderate obesity. Texas and Central California had counties with a high prevalence of moderate but not extreme obesity.

Hot spots identified for each obesity group were partially consistent with previous research of patterns among overall obesity, although earlier studies identified either smaller hot spots or additional ones.^32^ For example, Slack et al^20^ found a hot spot in an area that spanned the border between North Dakota and South Dakota that was not identified among either obesity group in this study. Still, findings from the present study seem to indicate that the hot spot in these states may be associated specifically with higher rates of extreme obesity; this area was identified as a high-low area in the local indicators of spatial association results for extreme obesity and was surrounded by a large cold spot. That is, there was an outlier county with high prevalence of extreme obesity surrounded primarily by counties with low prevalence.

During model construction, rurality was not found to be significantly associated with extreme obesity in the adjusted model, which was unexpected given the general association found in the literature.^21,33^ One possibility for this finding is that the variation often identified by rurality was more strongly accounted for by other sociodemographic indicators. Still, cold spots of low prevalence were found in metropolitan regions of states traditionally identified to have high prevalence of obesity: Atlanta, Georgia, and metropolitan areas in Texas were small cold spots for extreme obesity and, to a lesser extent, moderate obesity. These findings are consistent with obesity research indicating lower rates in urban relative to rural areas.^21^

Area-level poverty was also not found to be significantly associated with extreme obesity in the adjusted model, although differences by sex and race/ethnicity have been identified in the association between socioeconomic status and obesity, with a positive association found among women and a varying pattern of association among men of differing race and ethnicities.^34^ Future analyses using small-area estimates should explore extreme obesity prevalence stratified by sex.

### Limitations

There were a few limitations of this study. The BRFSS relies on self-reported weight and height to calculate BMI, which has been demonstrated to underestimate BMI with a greater bias among those with obesity and showing variability by demographic factors, including race and ethnicity.^35,36^ Estimates are conservative with true rates of extreme and moderate obesity likely higher than those based on self-reports. Prevalence using surveyor-measured weight and height from 2011 to 2012 indicated a national prevalence of 6.4%.^3^ Several methods have been proposed for correcting self-reported BMI, yet there is no consensus on the best approach.^37,38,39^ Because individuals with high BMI are more likely to underreport weight, the issue of underestimation may be more severe in this analysis than in others focusing on general obesity, further highlighting the need for action. In addition, it would be ideal to assess trend over time and examine more recent estimates of extreme obesity. The most recently available national estimates using surveyor-measured weight and height found that extreme obesity increased 20% as of 2015-2016 (prevalence, 7.7%).^3^ At this time it is not possible to create estimates for BRFSS surveys after 2012 as more recent waves lack a county indicator in the publicly available data sets. However, differences in prevalence of extreme obesity by age, sex, and race/ethnicity—the main covariates used in the prediction model—have remained consistent over time,^1,2,3,40^ which indicates a continued relevance to variation in estimates obtained.

A concern with small-area estimates is that it is difficult to externally validate findings, although correlation methods were used in this research to assess internal validity.^41^ Still, surveillance data that allowed for direct estimation would be preferable. Federal and local government agencies should devote resources to better track obesity and other obesity-related chronic health conditions.

Despite these limitations, this study provides informative data about extreme obesity, which has been described as an integral component of the weight distribution in the US^7^ and has a deleterious effect on life expectancy that is comparable to the effect of cigarette smoking.^42^ Future analyses with access to more recent data could examine temporal changes in prevalence rates, particularly to monitor counties with above-average increases in rates and determine whether demographic changes in counties result in changes in prevalence rates.

## Conclusions

The findings of this study suggest that MRP can be used with available individual-level and county-level indicators to generate county-level estimates of extreme and moderate obesity and that results show apparently substantial and informative variation in county-level prevalence rates. While this technique requires several steps, including data manipulation and an understanding of multilevel modeling, the estimates produced can be useful for understanding a more localized variation in prevalence rates. Extreme obesity poses a sizable health burden in many areas of the US and integrated, comprehensive, and wide-reaching solutions are needed for population-based approaches to prevention and treatment. These efforts should include national, state, and local (county and municipal) approaches aiming to implement a broad spectrum of policies, ranging from health promotion (eg, healthy eating and physical activity) to systemic approaches, such as the currently debated legislation regarding the Treat and Reduce Obesity Act, which seeks to expand insurance coverage for obesity treatment.^43^

## Notes

Supplement. **eMethods 1.** Description of Geographic Aggregation **eMethods 2.** Description of the Multilevel Regression Model **eMethods 3.** Description of Poststratification **eFigure 1.** Flowchart Describing the Process of Linking Data From Multilevel Regression and Poststratification **eMethods 4.** Description of the Global and Local Moran’s *I* Indicators **eFigure 2.** Sensitivity Analysis Comparing Results From Local Moran’s *I* Using Queen, Rook, and k-Nearest Neighbor Weights **eTable.** Weighted Prevalence of Extreme and Moderate Obesity by Individual-Level Model Covariates Used for Estimating Predicted Risk, United States, BRFSS 2012 **eFigure 3.** Box-Plot Distribution of County-Level Prevalence of Extreme Obesity by State **eFigure 4.** Box-Plot Distribution of County-Level Prevalence of Moderate Obesity by State
