# Supplementary material for: Use of Small-Area Estimates to Describe County-Level Geographic Variation in Prevalence of Extreme Obesity Among US Adults
Source: JAMA Netw Open. 2020 May 8;3(5):e204289. doi: 10.1001/jamanetworkopen.2020.4289 (PMC7210484; doi:10.1001/jamanetworkopen.2020.4289)
Supplement: Supplement. — eMethods 1. Description of Geographic Aggregation eMethods 2. Description of the Multilevel Regression Model eMethods 3. Description of Poststratification eFigure 1. Flowchart Describing the Process of Linking Data From Multilevel Regression and Poststratification eMethods 4. Description of the Global and Local Moran’s I Indicators eFigure 2. Sensitivity Analysis Comparing Results From Local Moran’s I Using Queen, Rook, and k-Nearest Neighbor Weights eTable. Weighted Prevalence of Extreme and Moderate Obesity by Individual-Level Model Covariates Used for Estimating Predicted Risk, United States, BRFSS 2012 eFigure 3. Box-Plot Distribution of County-Level Prevalence of Extreme Obesity by State eFigure 4. Box-Plot Distribution of County-Level Prevalence of Moderate Obesity by State [file jamanetwopen-3-e204289-s001.pdf]

Supplementary Online Content

Mills CW, Johnson G, Huang TTK, Balk D, Wyka K. Use of small area estimates to describe county level geographic variation in prevalence of extreme obesity among US adults. *JAMA Netw Open*. 2020;3(5):e204289. doi:10.1001/jamanetworkopen.2020.4289

eMethods 1. Description of Geographic Aggregation

eMethods 2. Description of the Multilevel Regression Model

eMethods 3. Description of Poststratification

eFigure 1. Flowchart Describing the Process of Linking Data From Multilevel Regression and Poststratification

eMethods 4. Description of the Global and Local Moran's *I* Indicators

eFigure 2. Sensitivity Analysis Comparing Results From Local Moran's *I* Using Queen, Rook, and k-Nearest Neighbor Weights

eTable. Weighted Prevalence of Extreme and Moderate Obesity by Individual-Level Model Covariates Used for Estimating Predicted Risk, United States, BRFSS 2012

eFigure 3. Box-Plot Distribution of County-Level Prevalence of Extreme Obesity by State

eFigure 4. Box-Plot Distribution of County-Level Prevalence of Moderate Obesity by State

This supplementary material has been provided by the authors to give readers additional information about their work.

© 2020 Mills CW et al. *JAMA Network Open.*

eMethods 1. Description of Geographic Aggregation

Aggregation was conducted using the Geographic Aggregation Tool (GAT) R version 1.33. A BRFSS dataset with complete cases to be used in the model was created; respondents missing BMI, age, race/ethnicity, or county indicator were removed. Variable summaries by county (FIPS5) were run for total observations and ultimately saved as a shapefile, which was imported into the data aggregation tool. Several levels of aggregation were explored using the tool: total count = 5, 10, 25; allowing and restricting counties to merge across states. A determination was made by visual inspection of the new geographies to combine areas only when needed to ensure representation for each area. Because a small area estimation approach was used after this step to create prevalence estimates, it was not necessary to obtain geographical regions with enough observations to produce stable estimates. The final aggregation setting used required each area contained 5 observations. The determination to restrict counties from merging across states was made both because of the conceptualization of counties nested within states in the multilevel models and to facilitate the internal validation technique that compared state-level estimates.

In 2012, there were 3,109 counties within the 48 states in the contiguous United States and Washington, D.C. After county aggregation using the Geographical Aggregation Tool, there were 2,215 county or county-like areas. Of the 430 aggregations, 57.4% included only two counties and 90% of aggregations contained four or fewer counties.

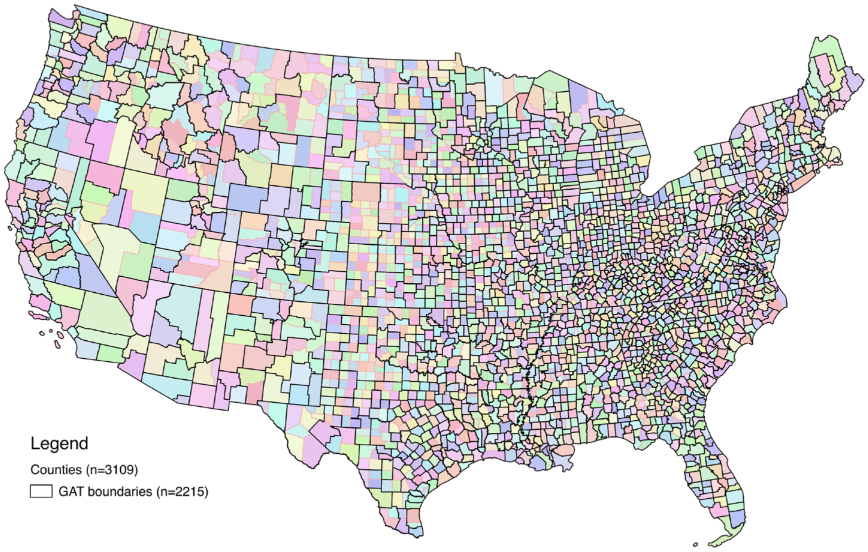

Reference

Talbot TO, LaSelva GD. *Geographic Aggregation Tool*. Albany, NY: New York State Health Department; 2015. https://www.albany.edu/faculty/ttalbot/GAT/GAT_vR13_guide.pdf.

© 2020 Mills CW et al. *JAMA Network Open.*

eMethods 2. Description of the Multilevel Regression Model

Final adjusted models were specified as follows:

$$P(y_{ijk} = 1) = logit^{-1}\left(\beta_{000} + b_{1jk}age_{ijk} + b_{2jk}sex_{ijk} + b_{3jk}race_{ijk} + b_{01k}edu_{jk} + b^{*}_{00k} + b^{*}_{0jk}\right)$$

Allowing i to refer to the individual, j to the county, and k to the state, the probability of extreme obesity is modeled by the inverse logit of intercept β_{000}, with b_{1jk}representing the regression coefficient for individual age group, b_{2jk} representing the coefficient for individual sex, b_{3jk} representing the coefficient for individual race/ethnicity, and b_{01k} representing the regression coefficient for the county education quartile. The adjustment to the error term due to state as a random effect (state-dependent deviation) is referred to by b^{*}_{00k} and the adjustment due to county as a random effect by (county-dependent deviation) b^{*}_{0jk}. A similar model was constructed modeling the probability of moderate obesity.

© 2020 Mills CW et al. *JAMA Network Open.*

eMethods 3. Description of Poststratification

This can be depicted as:

$$P^{c}_{lmncs} \times Pop^{b}_{lmncs}$$

where c indicates county and P^c is the predicted probability of extreme obesity for an individual in age l, sex m, race/ethnicity category n in county c and state s. Pop^{b}_{lmncs} represents each county's subgroup's respective population count.

After weighting the predicted value by the actual frequency of each sex-age-race/ethnicity cell within each county for each of the aforementioned cells, all cells within a county were summed to produce the county-level prevalence estimates.

$$P^c = \frac{\sum_l \sum_m \sum_n (P^{c}_{lmncs} \times Pop^{b}_{lmncs})}{\sum_l \sum_m \sum_n (Pop^{b}_{lmncs})}$$

$$= \frac{\sum_l \sum_m \sum_n (P^{c}_{lmncs} \times Pop^{b}_{lmncs})}{Pop^c}$$

References

Zhang X, Holt JB, Lu H, et al. Multilevel Regression and Poststratification for Small-Area Estimation of Population Health Outcomes: A Case Study of Chronic Obstructive Pulmonary Disease Prevalence Using the Behavioral Risk Factor Surveillance System. *Am J Epidemiol*. 2014;179(8):1025-1033. doi:10.1093/aje/kwu018

Pacheco J. Using National Surveys to Measure Dynamic U.S. State Public Opinion: A Guideline for Scholars and an Application. *State Politics & Policy Quarterly*. 2011;11(4):415–439.

© 2020 Mills CW et al. *JAMA Network Open.*

eFigure 1. Flowchart Describing the Process of Linking Data from Multilevel Regression and Poststratification

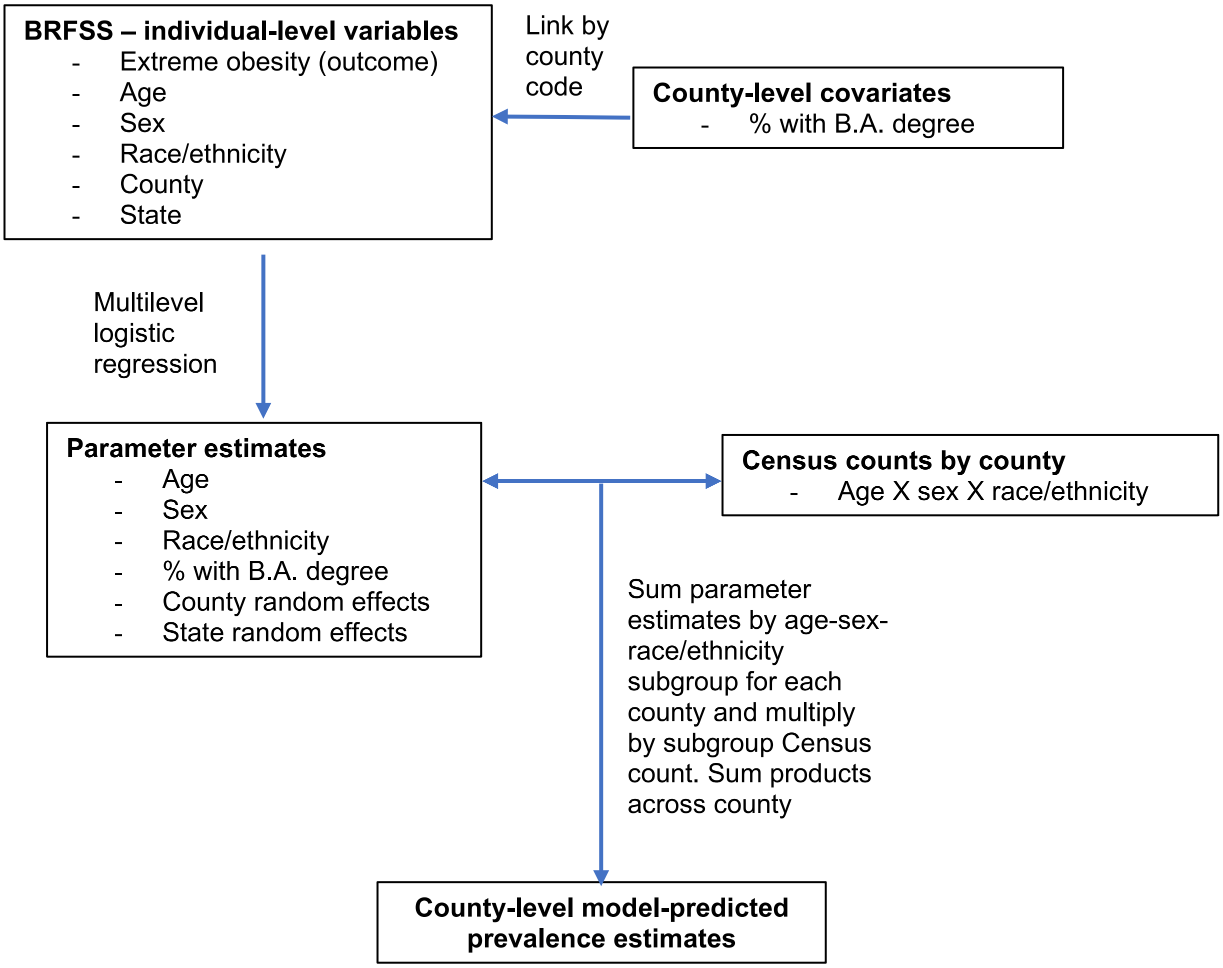

Data sources: 2012 BRFSS, 2012 ACS 5-year roll-up (county-level covariates); and 2010 US Census

© 2020 Mills CW et al. *JAMA Network Open.*

eMethods 4. Description of the Global and Local Moran's *I* Indicators

The Moran's I is defined as:

$$I = \frac{n}{S_0} \frac{\sum_{i=1}^{n} \sum_{j=1}^{n} w_{ij} z_i z_j}{\sum_{i=1}^{n} z_i^2}$$

for observations at $i = 1, \ldots, n$ counties, z_i and z_j represents the deviations from the country-wide mean, $(x_i - \bar{x})$ and $(x_j - \bar{x})$ respectively, and w_{ij} is the weight assigned to all j counties, relative to their spatial adjacency to county i, as detailed in eFigure2. S_0 represents the aggregate of all spatial weights and is given by:

$$S_0 = \sum_{i=1}^{n} \sum_{j=1}^{n} w_{ij}$$

Using notation similar to the global Moran's I, the local indicators can be depicted as follows:

$$I_i = z_i \sum_j w_{ij} z_j$$

Such that I_i refers to the local Moran's coefficient at county i and deviations from the regional mean are summed over j neighboring values. We determined significance for counties within geographical clusters based on how far the local Moran's Index deviates from the Monte Carlo simulated distribution under the null hypothesis of complete spatial randomness – that is, significantly higher or lower prevalence of extreme obesity than would be expected, using a threshold of $p<0.05$.

Reference

Anselin L. Local indicators of spatial association—LISA. *Geographical analysis*. 1995;27(2):93-115.

eFigure 2. Sensitivity Analysis Comparing Results from Local Moran's *I* Using Queen, Rook, and k-Nearest Neighbor Weights

A weights matrix defines the spatial relationships such that closer areas receive a greater weight in calculations than those that are further away. In the queen matrix, all areas contiguous to the observed location are incorporated into the weighting scheme (as in a queen's movement in chess). In a rook matrix, regions are spatial neighbors if they share a side but not if they meet at only one vertex. For a k-nearest neighbors' matrix, centroid distances between polygons are calculated to determine the closest k neighbors for incorporation into the weighting scheme. A first-order queen contiguity weights matrix was chosen, though a sensitivity analysis was run utilizing both a rook and k-nearest neighbors (k=4) matrix.

Extreme obesity

Weight: Queen contiguity, order of contiguity=1
Moran's I: 0.352, z-value: 28.230, *p* value: 0.001

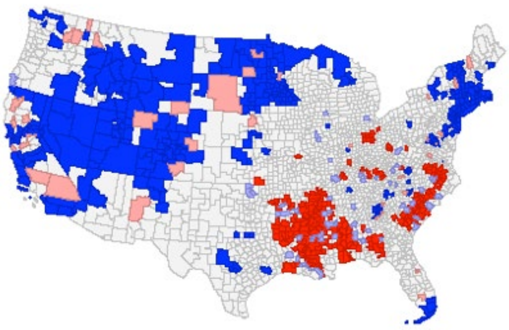

Weight: Rook contiguity, order of contiguity=1
Moran's I: 0.356, z-value: 27.900, *p* value: 0.001

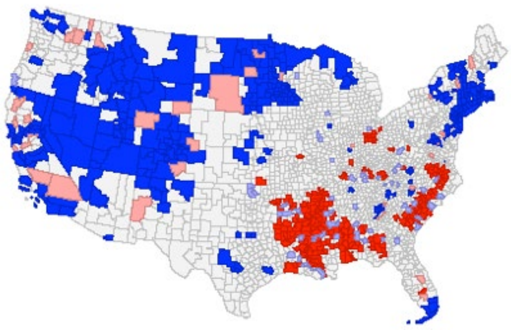

Weight: k-Nearest Neighbors, number of neighbors=4
Moran's I: 0.3518, z-value: 24.560, *p* value 0.001

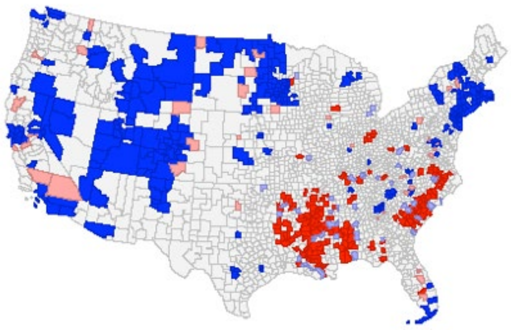

© 2020 Mills CW et al. *JAMA Network Open.*

(con't) Moderate obesity

Weight: Queen contiguity, order of contiguity=1
Moran's I: 0.376, z-value: 29.504, *p* value: 0.001

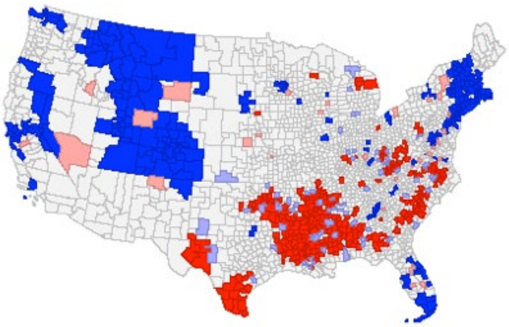

Weight: Rook contiguity, order of contiguity=1
Moran's I: 0.381, z-value: 30.328, *p* value: 0.001

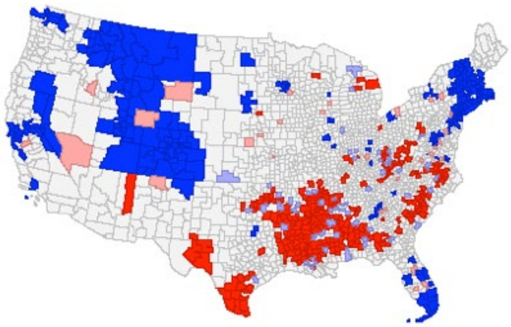

Weight: k-Nearest Neighbors, number of neighbors=4
Moran's I: 0.394, z-value: 25.915, *p* value: 0.001

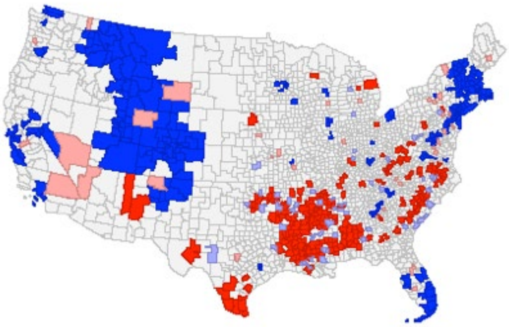

© 2020 Mills CW et al. *JAMA Network Open.*

eTable. Weighted Prevalence of Extreme and Moderate Obesity by Individual-Level Model Covariates Used for Estimating Predicted Risk, United States, BRFSS 2012*

	Extreme obesity			Moderate obesity		
**Variable**	Prevalence (%)	95% CI		Prevalence (%)	95% CI	
Overall	4.0	3.9	4.1	23.7	23.4	23.9
Sex						
Male	3.2	3.0	3.3	24.8	24.5	25.2
Female	4.9	4.7	5.0	22.5	22.1	22.8
Age group, years						
18-24	2.2	1.9	2.5	12.8	12.1	13.6
25-29	3.6	3.1	4.1	20.1	19.1	21.1
30-34	4.7	4.2	5.2	22.7	21.8	23.6
35-39	5.4	4.8	5.9	25.8	24.8	26.9
40-44	5.2	4.7	5.6	26.2	25.3	27.2
45-49	4.7	4.3	5.1	27.3	26.4	28.3
50-54	4.7	4.3	5.1	28.0	27.1	28.8
55-59	4.9	4.5	5.3	28.3	27.5	29.2
60-64	5.2	4.8	5.6	28.2	27.3	29.0
65-69	3.8	3.5	4.2	28.0	27.2	28.9
70-74	3.0	2.6	3.4	26.0	25.1	26.9
75-79	1.9	1.5	2.4	22.1	21.2	23.1
80 and older	0.7	0.6	0.9	15.3	14.3	16.2
Race/ethnicity						
White	3.6	3.5	3.8	22.8	22.6	23.1
Black	7.6	7.0	8.1	30.2	29.2	31.1
Hispanic	3.9	3.5	4.3	26.4	25.4	27.4
Asian	0.7	0.3	1.0	9.1	7.8	10.4
Hawaii Native/Pacific Islander	3.5	1.5	5.4	22.4	17.4	27.4
American Indian/Alaska Native	5.2	4.2	6.3	28.5	26.3	30.8
Other race	2.6	1.8	3.4	21.6	18.9	24.3
Multiracial	4.1	3.3	4.8	25.6	23.5	27.7

*Includes data from all 50 states and Washington, D.C.

© 2020 Mills CW et al. *JAMA Network Open.*

eFigure 3. Box-Plot Distribution of County-Level Prevalence of Extreme Obesity by State

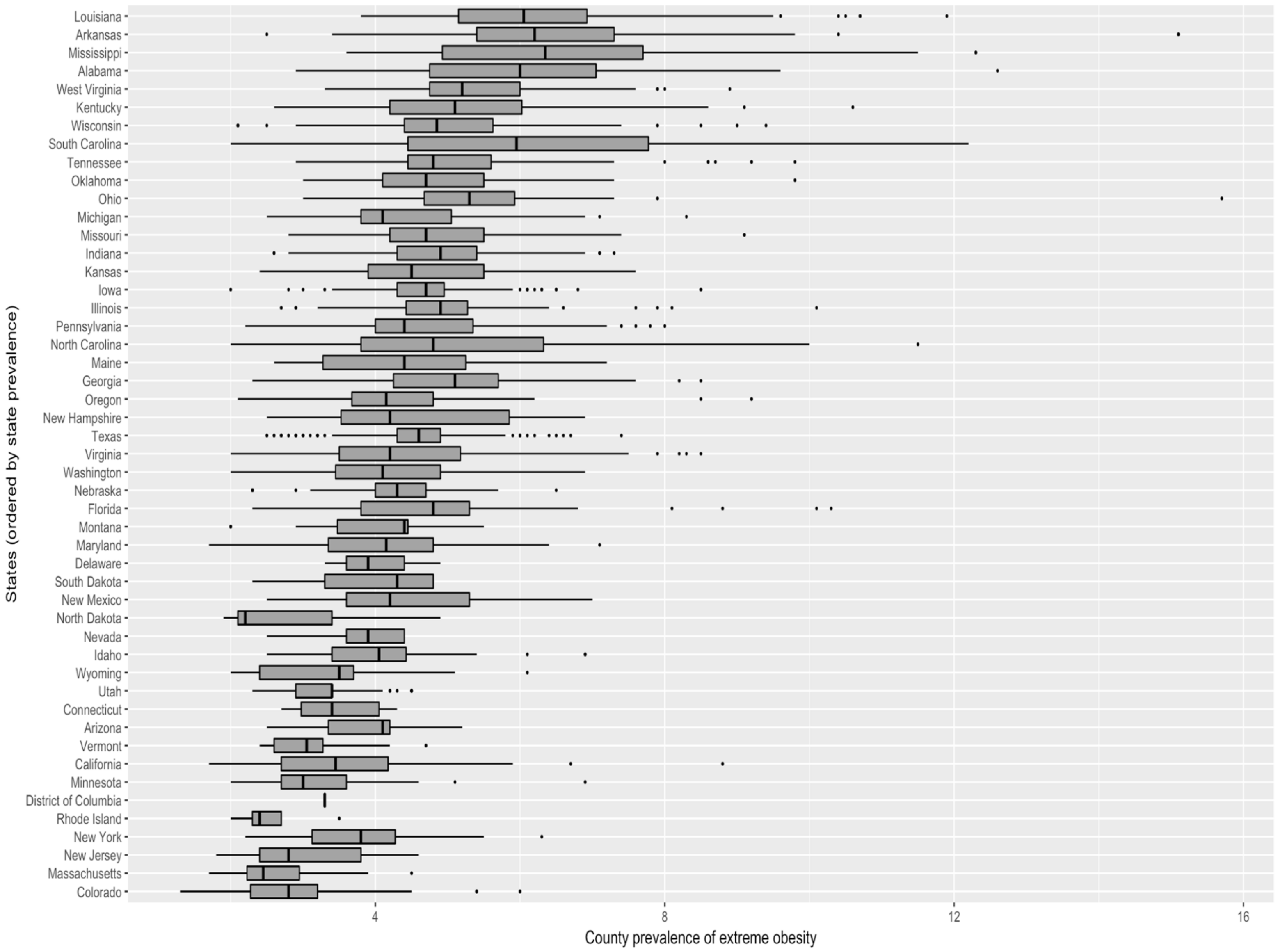

© 2020 Mills CW et al. *JAMA Network Open.*

eFigure 4. Box-Plot Distribution of County-Level Prevalence of Moderate Obesity by State

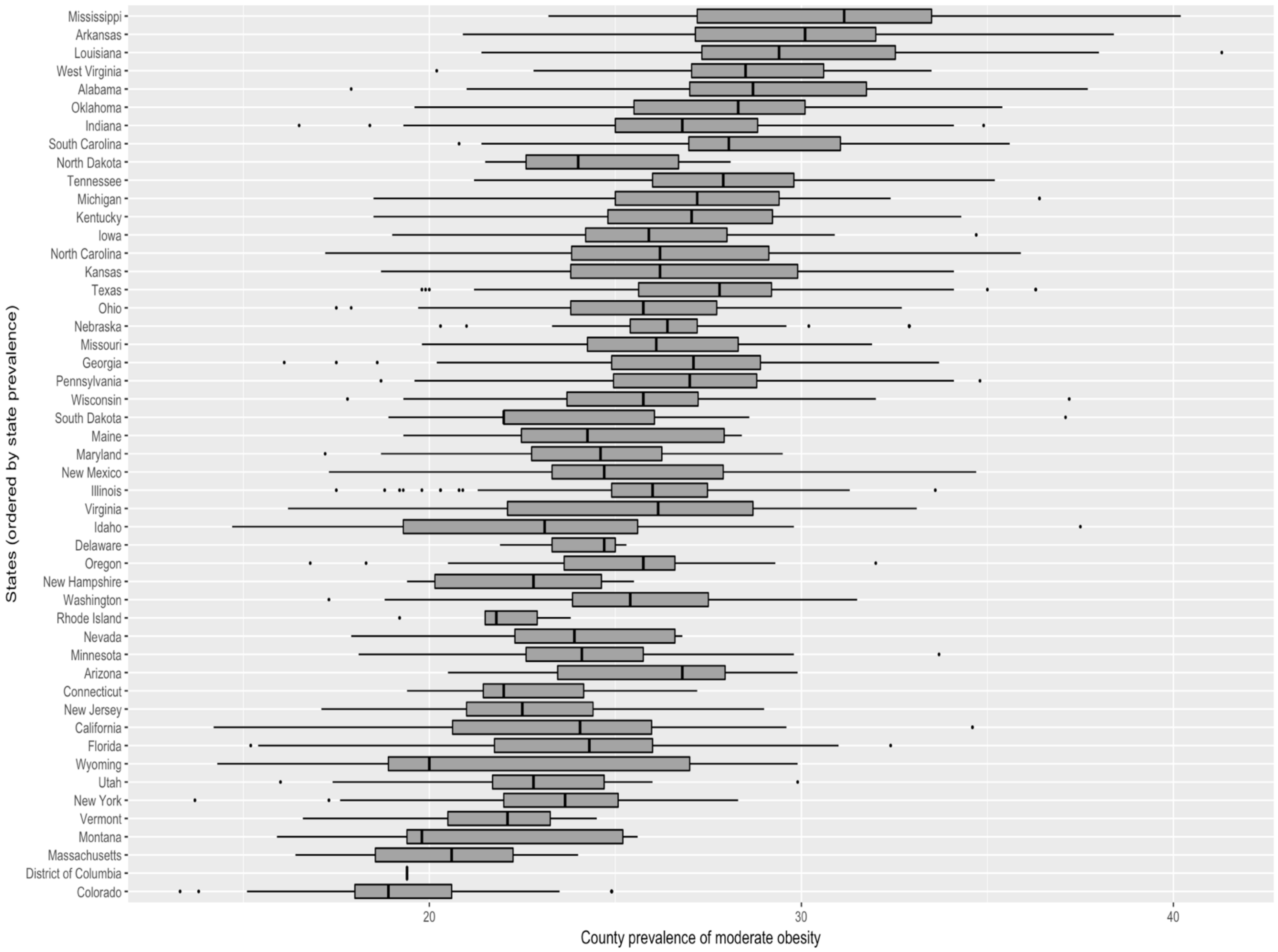

© 2020 Mills CW et al. *JAMA Network Open.*
